# Natural Killer T-like Cells: Immunobiology and Role in Disease

**DOI:** 10.3390/ijms24032743

**Published:** 2023-02-01

**Authors:** Jani-Sofia Almeida, José Manuel Casanova, Manuel Santos-Rosa, Raquel Tarazona, Rafael Solana, Paulo Rodrigues-Santos

**Affiliations:** 1 Institute of Immunology, Faculty of Medicine, University of Coimbra (FMUC), 3004-504 Coimbra, Portugal; 2 Laboratory of Immunology and Oncology, Center for Neuroscience and Cell Biology (CNC), University of Coimbra, 3004-504 Coimbra, Portugal; 3 Center of Investigation in Environment, Genetics and Oncobiology (CIMAGO), Faculty of Medicine, University of Coimbra, 3004-504 Coimbra, Portugal; 4 Coimbra Institute for Clinical and Biomedical Research (iCBR), Faculty of Medicine, University of Coimbra, 3000-548 Coimbra, Portugal; 5 Center for Innovation in Biomedicine and Biotechnology (CIBB), University of Coimbra, 3004-504 Coimbra, Portugal; 6 Clinical Academic Centre of Coimbra (CACC), 3000-075 Coimbra, Portugal; 7 University Clinic of Orthopedics, Orthopedics Service, Tumor Unit of the Locomotor Apparatus (UTAL), Coimbra Hospital and Universitary Center (CHUC), 3000-075 Coimbra, Portugal; 8 Immunology Unit, Department of Physiology, University of Extremadura, 10003 Cáceres, Spain; 9 Maimonides Biomedical Research Institute of Cordoba (IMIBIC), Reina Sofía University Hospital, 14004 Córdoba, Spain; 10 Immunology Unit, Department of Cell Biology, Physiology and Immunology, University of Córdoba, 14071 Córdoba, Spain

**Keywords:** natural killer T-like cells, innate-like cells, ageing, inflammation, infection, pregnancy, transplantation, autoimmunity, neurological disorders, cancer

## Abstract

CD56+ T cells are generally recognized as a distinct population of T cells and are categorized as NKT-like cells. Although our understanding of NKT-like cells is far from satisfactory, it has been shown that aging and a number of disease situations have impacted these cells. To construct an overview of what is currently known, we reviewed the literature on human NKT-like cells. NKT-like cells are highly differentiated T cells with “CD1d-independent” antigen recognition and MHC-unrestricted cell killing. The genesis of NKT-like cells is unclear; however, it is proposed that the acquisition of innate characteristics by T cells could represent a remodeling process leading to successful aging. Additionally, it has been shown that NKT-like cells may play a significant role in several pathological conditions, making it necessary to comprehend whether these cells might function as prognostic markers. The quantification and characterization of these cells might serve as a cutting-edge indicator of individual immune health. Additionally, exploring the mechanisms that can control their killing activity in different contexts may therefore result in innovative therapeutic alternatives in a wide range of disease settings.

## 1. Introduction

The immune system is a complex and dynamic organization that coordinates the organism’s immunosurveillance and defense [1]. Immune cells are traditionally classified into two groups: innate cells that are “ready to use” and adaptive cells that need to be primed to recognize the target cells. Despite the practical division of innate and adaptive cell populations, cells from both families communicate and depend on one another to be functional. The neural cell adhesion molecule (NCAM), also known as CD56, is considered a distinctive marker of human NK cells, although it has been observed that other innate and adaptive immune cells may eventually upregulate or neo-express this molecule [2,3]. 

In NK cells, the CD56 molecule promotes the formation of strong synapses with stromal cells that ultimately leads to NK cell maturation [4]. Apart from NK cells, it is not known why other immune cells acquire the expression of the CD56 molecule and what is the objective of this uncommon expression. It is important to be aware that CD56 expression is highly interspecies dependent, which is critical when comparing studies in different species [5]. For instance, rodents do not express the CD56 molecule; instead, they express other molecules used as NK cell markers, including DX5/CD49b, NK1.1, and NKR-P1A.

In vivo, the expression of the CD56 molecule may occur on different subsets of human T cells and has been associated with increased cytotoxic potential [6]. The CD56 molecule can be expressed by “conventional” T cells that recognize peptides bound to classical major histocompatibility complex (MHC) molecules, such as cytotoxic CD8 T cells, and these cells often express other NK-related receptors (NKR) [7]. In a similar way, the expression of CD56 on T cells, accompanied by other NKR, is a common characteristic of “unconventional” T cells that recognize antigens via MHC class I-b and MHC class I-like molecules [8,9]. NKT cells (CD1-restricted T cells), MAIT cells (mucosal-associated invariant T cells), and γδ T cells are unconventional T cells and may or may not express the CD56 molecule [6,8,10]. In vitro, peripheral blood mononuclear cells (PBMC) maintained in long-term cultures with IL-2 promote the expansion of clones that express simultaneously the CD3 and the CD56 molecules, and these clones exhibit the highest killing potential against tumor cells [11,12,13,14].

The CD56+ T cells are generally recognized as a distinct population of T cells and are categorized as NKT-like cells. Although our understanding of NKT-like cells is far from satisfactory, it has been shown that aging and a number of disease situations have impacts on these cells [15,16]. Therefore, we reviewed the literature on human NKT-like cells to compile an overview of what is currently known about their function in both health and disease.

## 2. NKT-like Cells: What’s in a Name?

Given the conflicting literature on NKT and NKT-like cells, it is important to first establish that NKT-like cells are not represented by the most extensively studied populations of NKT cells. 

NKT cells were first defined in mice as T cells that express NK cell receptors [17]. However, this definition commonly refers to CD1d-restricted, lipid antigen–reactive T lymphocytes that can increase cell-mediated immunity to infectious organisms and malignancies but can also decrease cell-mediated immunity associated with autoimmune illness and allograft rejection [18,19,20]. In mice, type I NKT cells, or invariant NKT (iNKT), cells were discovered as a distinct T cell population expressing the Vα14Jα18 invariant TCR-chain. When presented via MHC class I-like CD1d molecules, the glycosphingolipid -galactosylceramide (-GalCer) or its synthetic analogs were recognized by the invariant TCR expressed on the type I NKT cell subset. A few years later, the Vα24Jα18 human homolog was found, which primarily couples with the Vβ11 TCR chain. Type II NKT cells differ from type I NKT cells in that they have a more varied and less well-defined TCR repertoire that recognizes non-GalCer molecules (mainly sulfatide) provided by CD1d molecules. As the classic NKT family is beyond the scope of the present review, we would like to direct readers to Godfrey et al. for a comprehensive examination [18,21]. 

### 2.1. Definition of NKT-like Cells

In 1986, a population of cells sharing the expression of the CD3 (T3) and CD56 (NKH1 or Leu-19) molecules was reported for the first time [22]. Those cells were defined as a population of large granular lymphocytes expressing both CD3 and CD56, with azurophilic granules capable of killing “NK-sensitive” tumor cell targets in a non-MHC-restricted manner. In 1995, the denomination “NK-T cell” was used to identify a subgroup of mouse T cells that shared some characteristics with NK cells, and, particularly, the expression of the NK1.1 marker (Nkrp1c or CD161c) was first reported [17]. The fact that most regularly used mouse strains does not express the NK1.1 marker has long confounded the classification of NKT cells [23]. Thus, it is clear that the simplified characterization of NKT cells as NK1.1+ T cells is not only inaccurate but also potentially misrepresentative [18]. 

The existence of NKT cell subsets and other types of T cells that resemble NKT cells, is a source of confusion in the literature. However, researchers have recently sought clarification on this topic, and it is now widely agreed that some cells are included in the NKT-cell umbrella while others should be considered distinct, such as NKT-like cells [16,21]. Thereby, the definition of NKT-like cells (CD3+CD56+) was raised to clearly distinguish those cells from the classical NKT family. Even though some overlap can be observed with these two types of populations, NKT-like cells represent highly differentiated, conventional, or unconventional T cells that co-express the CD56 molecule and other NKR and recognize antigens in a “CD1d-independent” manner.

### 2.2. Phenotype

NKT-like cells represent less than 10% of circulating lymphocytes in adulthood and exhibit a high-density TCR-CD3 complex, similar to classical T cells, and the low density of the CD56 molecule, similar to cytotoxic NK cells [24]. Regardless the evident heterogeneity of NKT-like cells is the higher proportion of cells corresponding to classical αβ TCR+ CD8 T cells [7], although there are also reports of αβ TCR+ CD4 T cells [25]. It is known that NKT, MAIT, and γδ T cells may also express the CD56 molecule; thus, when studying CD56+ T cells, a mixture of conventional and unconventional T cells can be found [6,10]. 

Phenotypically and morphologically, NKT-like cells are highly comparable with cytotoxic NK cells, except for the expression of the TCR-CD3 complex, the low or absent expression of CD16, and the higher expression of the IL-2 receptor-beta chain (IL-2Rβ) [26]. NKT-like cells also express natural cytotoxic receptors (NCR) such as NKp44 and NKp46 and activation-related markers such as CD69 and HLA-DR [7,24]. NKT-like cells express the same NKR, costimulatory, cytokine, and chemokine receptors as iNKT cells [7,27]. The exceptions include the killer-cell immunoglobulin-like receptors (KIR), commonly found on NK cells, which upon interacting with the human leukocyte antigen (HLA) molecules, promote either inhibitory or stimulatory signals [28,29]. Chan et al. observed the higher frequency of KIR+ cells within the CD56+ compartment and observed a high intensity for DNAM-1 expression [7]. Furthermore, the examination of the metabolic transcriptome demonstrates that KIR+CD56+ T cells are immunological memory cells, as opposed to KIR−CD56+ T cells, which are more active in energy metabolism and effector differentiation.

NKRs are also expressed by a fraction of CD8+ T cells in mice and humans, and these cells have a memory phenotype [30]. Comparatively to their CD56− counterparts, mucosal CD56+ T cells show a reduced capacity to proliferate, which is typical of their mature state [31]. Romero et al. identified several CD56+ T cell phenotypes, revealing a previously unappreciated heterogeneity among these cells [24]. A phenotypical continuum, which is partially reflected by the unique CD3+CD56+ subset description, consists of phenotypes layered with naïve, memory, type 1, 2, and 17 differentiation stages. 

Cytomegalovirus (CMV) infection is associated with NK cells’ increased expression of NKG2C and CD57 [32,33]. These NKG2C+CD57+ NK cell clones have been hypothesized to represent “memory-like” NK cells that have grown in response to infection. The expression of NKG2C was appreciated on CD56− and CD56+ T cells [34,35]. NKG2C expression in both populations correlates with highly differentiated T cells since they express the CD45RA molecule that is characteristic of the effector memory re-expressing CD45RA T cells. Particularly, NKG2C+CD56+ T cells exhibit a higher expression of NKG2D, CD16, KIR2DL2/L3, and the maturation marker CD57 than NKG2C+CD56− T cells. It was also shown that CMV infection led to the expansion of CMV-specific CD45RA+ memory CD8 T cells with an increased effector function and low expression of the CD28 molecule [36]. Both the development of CD56 and TCR-independent activity were linked with CD28 deletion: a characteristic of terminally differentiated CD8 T cells implicated in immunosenescence [10,37].

**Figure 1:**
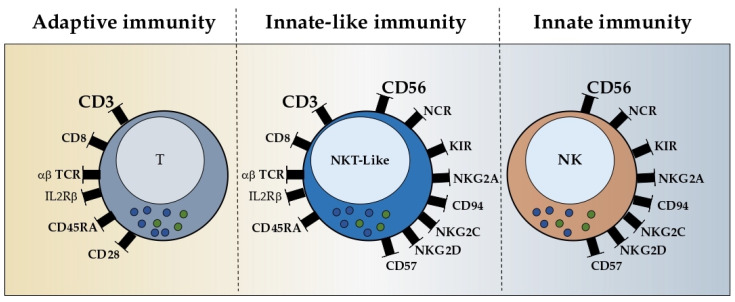
Receptor repertoire of NKT-like cells. NKT-like cells are T cells that acquire the expression of the CD56 molecule, as a typical marker of NK cells. In the middle of the image, it the receptor repertoire of NKT-like cells is presented, to the left, the receptor repertoire of T cells that are common to NKT-like cells is shown, and to the right, the receptor repertoire of NK cells that are common to NKT-like cells can be seen. T and NK cells are categorized as having an adaptive and innate immunity, respectively, whereas NKT-like cells are categorized with innate-like immunity, since NKT-like cells share mechanisms from both T and NK cells. The mature phenotype of NKT-like cells (CD45RA and CD57) includes a number of activating and inhibitory receptors (NCR, KIR, NKG2A/C/D, αβ TCR and IL2Rβ). NKT-like cells also have a high capacity for cytokine production and cytotoxicity. Blue and green circles represent granules and cytokines, respectively. TCR—T cell receptor; NCR—natural cytotoxic receptor; KIR—killer-cell immunoglobulin-like receptor.

Even though NKT-like cells are a very heterogeneous population, these cells share at least three characteristics, the mature phenotype, NKR acquisition, and granular morphology (Figure 1). Moreover, the most frequent population expresses the CD8 co-receptor and the αβ TCR. It is crucial to know whether these cells develop as an independent origin or as an adaptation of the pre-existing T cells to the microenvironment.

## 3. NKT-like Cells: Extrathymic Differentiation and Correlation with Aging

Although the origin of NKT-like cells is not entirely understood, extrathymic differentiation has been proposed. In addition to T cells differentiated in the thymus (TCR of bright intensity, TCR^bright^) and found in circulation, extrathymically differentiated T cells have been found in the liver and gut (TCR of intermediate intensity, TCR^int^) of mice [38,39]. Thereafter, TCR^int^ cells increased in the liver, whereas TCR^bright^ cells increased in the periphery as a function of age [40]. Extrathymic murine T cells are NK1.1+CD3+, categorized morphologically as large granular lymphocytes, and are able to mediate spontaneous cytotoxicity against tumor cells [41,42,43]. Human T cells that co-express the NK cell marker CD56 also increase with age and shares properties with murine TCR^int^ cells [44,45], supporting the idea that human NKT-like cells may also be of extrathymic origin, resembling TCR^int^ cells found in the liver and gut of mice [46].

### 3.1. Extrathymic Differentiation

In humans, there are two main groups of extrathymically differentiated T cells. Liver T cells express the IL-2Rβ constitutively and have a TCR^int^, intraepithelial and peripheral lymphocytes, a TCR of bright intensity, and a heterogeneous expression of IL-2Rβ [47]. 

CD56+ T cells make up roughly 50% of the liver T cells [48,49]. This suggests that hepatic CD56+ T lymphocytes can be primarily produced in the liver. In mice, a high proportion of liver CD56+ T cells are CD4−CD8−γδ or CD56+CD8+ αβ T cells, with a morphology of large granular lymphocytes [50]. In spite of the cytotoxic capacity of these cells, the liver shows a tolerogenic microenvironment [51]. The presence of these highly immunogenic T cells in immunotolerant organs, such as the liver, raises the possibility that they could co-evolve with suppressor cells and regulate them to control and maintain the aforementioned tolerogenic microenvironment [52].

The intestinal immune compartment is closely regulated, and its antigenic repertoire is separately developed [53]. CD56+ T cells and CD161+ T cells are present in the normal human colon and account for between 6.7% and 21.3% of all mononuclear cells, respectively [54]. The frequency of NKT-like cells is low in the small intestine of mice [55]. However, in the large intestine, there are a considerable number of NKT-like cells that are restricted by classic MHC or MHC-like molecules. Phenotypically, these cells are mostly CD8+ or CD4−CD8−. The human intestinal epithelium is the primary home of CD161+ T cells, and 50% of these cells express the CD56 molecule suggesting an important role in controlling gut immunity [56]. A sizeable portion of intestinal CD56+ T cells may have important immunoregulatory functions due to their strong effector potential and increased expression of proinflammatory cytokines. It was proposed that intestinal T cells may be linked to CD56+ T cells found in the peripheral blood [31]. Peripheral CD56+ T cells express gut-homing integrins, react to the gut-related CD2 signaling pathway, and share characteristics with mucosal T cells supporting that CD56 expression can identify circulating mucosal T lymphocytes with a putative effector function in mucosal immunity. As a result, the discovery of fully developed T lymphocytes expressing CD56 in the adult gut is intriguing and in line with the theory of differentiation independent of the thymus [31,57]. 

According to the extrathymic differentiation theory, it is most likely that these cells evolved in the liver and gut. Additionally, it is possible that the gut is the source of circulating NKT-like cells due to the resemblance between these cells and intraepithelial NKT-like cells, namely the intensity of the TCR expression. It has also been proposed that NKT-like cells may result in ongoing antigenic exposure because these localizations—the liver, the gut, and the peripheral circulation—are the ones with the highest frequency of NKT-like cells and are constantly exposed to antigens. The fact that the frequency of peripheral NKT-like cells rises with age also supports that ongoing antigen exposure may prompt NKT-like cell differentiation.

### 3.2. Age-Dependency

Studies in the 1980s revealed that T cells expressing the CD56 molecules are nearly absent in newborns [58,59]. Although they are barely undetectable in cord blood, they may be inducible from their precursors in vitro cultures, suggesting that the lifelong accumulation of these cells may be driven by antigenic experiencing [50]. Likewise, perforin-positive T lymphocytes are absent in cord blood, reaching significant proportions in adulthood [60]. 

It was reported that a considerable increase in the percentage of T cells in elderly people, including senior donors and centenarians, co-express various NK cell markers such as CD56, CD57, or CD94 differentiation antigens [61,62,63]. As the percentage of Vα24+ NKT cells in the elderly is not noticeably higher, the rise in this population of CD3+ cells that also express NK receptors is not attributable to the iNKT cell population [46]. It was postulated that the expression of NK receptors on CD8+ T cells could be viewed as a hallmark of cytotoxic effector T cells that are increased in vivo after antigenic stimulation leading to substantial proliferation. 

The loss of co-stimulatory molecules, telomere shortening, and poor IL-2 production brought on by antigen persistence defined the state of replicative senescence in T cells. Most of these “effector/senescent” T cells have the following phenotype: CD8+, CD45RA+, and CCR7− [15]. They are cytotoxic T cells that express intracytoplasmic perforin and granzyme B strongly; however, they have a poor ability to proliferate and produce IL-2. It was also suggested that the expression of NK receptors on T lymphocytes is the consequence of the accumulation of CD8+CD28− cytotoxic effector cells in response to persistent chronic activation, and their accumulation in vivo might be related to the resistance of these cells to apoptosis [15,64].

### 3.3. Successful Aging

The T cell immune response is probably the immune compartment that is most negatively affected by aging since the thymus, necessary for T cell priming and maturation, starts to involute right after your twenties [65]. It is also known that NK cells are sensitive to age-associated immunological changes [66,67]. Those successful among the elderly have a distinct T cell repertoire, characterized by the predominance of highly oligoclonal αβ T cells that express a wide variety of receptors often expressed by NK cells. 

NKT-like cells are functionally active effectors that do not require the activation of their clonotypic TCR while having cell senescence-related characteristics [68]. In contrast to exhausted T cells, senescent terminal effector memory differentiated CD45RA+ CD8+ T (TEMRA) cells are effector T cells with complete competence. TEMRA cells combat the increased burden of cancers and infections in the elderly by using their newly acquired NK cell machinery to sustain fast effector activities throughout life [69,70]. According to Pereira and Akbar, human CD8+ NKT-like cells are not abnormal; rather, they constitute a unique T cell population that compensates for the functional weaknesses of traditional NK and CD8+ T cells [69]. It was suggested that CD56+ T cells could mediate TCR-independent immunological cascades that may support protective immunity in the elderly [71]. The expression of the cellular protective proteins SIRT1, HSP70, and SOD2, which are implicated in the stress response, was examined in the T cells and NKT-like cells of the oldest seniors [72]. In all the examined age groups, NKT-like cells showed a noticeably higher expression of the cellular protective proteins HSP70 and SOD2 compared to T cells. Their expression and the individuals’ ages are correlated, and this event seems to be associated with longevity. 

**Figure 2:**
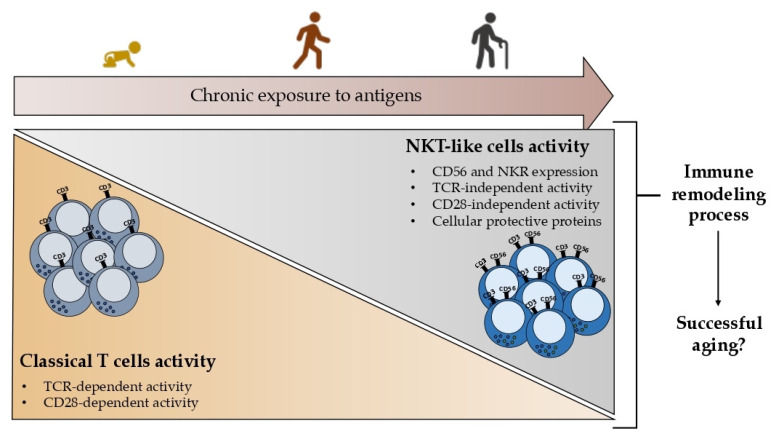
Illustration of the hypothesis that the age-related increase in NKT-like cells may result from long-term antigen exposure. In general, the TCR and CD28-dependent activity of classical T cells declines with age; conversely, NKT-like cells accumulate in the peripheral blood. Although the genesis of NKT-like cells is unknown, it has been hypothesized that the persistent antigen exposure of T cells may result in the acquisition of innate-like receptors, such as CD56. It is speculated that this mechanism, which is intimately tied to aging, occurs as an immunological remodeling process, and may assist or at the very least be associated with successful aging. Blue and green circles represent granules and cytokines, respectively.

Together these findings point out the importance of studying NKT-like cells in the context of aging since it is proposed that the acquisition of innate characteristics by T cells, namely the expression of the CD56 and other NKR, could represent a remodeling process leading to successful aging rather than a nonfunctional product of aging as represented in Figure 2.

## 4. NKT-like Cells: Properties

### 4.1. Expansion

After the initial studies about T cells expressing NKR, it was observed that IL-2 dependent T cell lines were maintained in long-term cultures and led to the expansion of cytokine-induced killer (CIK) cells expressing both CD3 and CD56 molecules, which are cytotoxic and may express the CD8 or CD4 co-receptor [73]. It was shown that IL-2 and IL-15 co-stimulatory cytokines induce the proliferation of CD56+ T cells, whereas this effect was not observed for subpopulations of CD56- T cells [74]. It was also demonstrated that IL-15 was clearly superior to IL-2 in its potential to expand and double the number of NKT-like and NK cells [75,76]. Additionally, the effect of IL-15 on the expansion of γδ T or classical CD8 T cells was previously reported[74]. Perhaps the CD8 T cells that expand with IL-2 are those expressing the CD56 molecule. IL-15-induced CD8+CD56+ T cells exhibited memory and activated phenotype, expressing CD45RO and CD69, respectively, and representing the most cytotoxic phenotype [76]. Furthermore, it was proposed that IL-15 stimulated the expression of CD56 on immune cells with the IL-2/IL-15R β chain, including NK and T cells [77]. 

The expansion of CD3+CD56+ clones using a protocol with IFN-γ, IL-1, IL-2, and anti-CD3 led to an increase of 6000-fold after two weeks of culture [78]. TNFR superfamily member CD137 (4-1BB) also mediates the costimulatory signal that causes T and NK cells to proliferate and produce cytokines. Anti-CD137 mAb was found to significantly enhance the proliferation of CD3+CD56+ CIK cells. The same was observed for PBMC [79]. 

Collectively, these results demonstrate that IL-15, IFN-γ, anti-CD3, and anti-CD137 induce the expansion of NKT-like cells, which mostly express the co-receptor CD8. In vitro cultures of PBMC showed an increase in the acquisition of CD56 expression by T cells, suggesting that NKT-like cells accumulating in vivo might be the result of expansion via cytokine exposure or activating receptor recognition.

### 4.2. Activation and Cytokine Production

When exposed to IL-2, IL-12, IL-15, and IL-18, both CD56+ T cells and NK cells have the ability to produce more IFN-γ than ordinary CD8+ T cells [80,81,82]. The production of IFN-γ by NKT-like cells and NK cells are also promoted using IL-12 and IL-18 in combination, whereas IL-23 and IL-18 only stimulate NKT-like cells [83]. Interestingly, IL-23 leads to the upregulation of CD56 on NKT-like cells. The activation of NK and NKT-like cells early in infection and the shaping of Th1 differentiation via IFN-γ were thought to be mediated by the antigen-presenting cell (APC)-derived IL-23.

Additionally to the production of IFN-γ, NKT-like cells, when activated, release higher amounts of TNF-α, IL-5, and IL-13 [84]. On the other hand, NKT-like cells barely produce any regulatory (IL-10) and T helper 2 (IL-4 and IL-5) cytokines [31]. When CIK cells were stimulated with anti-CD137, they produced more IFN-γ, IL-2, and TNF-α while producing less TGF-β, IL-4, and IL-10 [79]. Contrarily, when CD137 expression was inhibited in CD28^null^ T cells and NKT-like cells, IFN-γ, TNF-α, and granzyme B production were down-regulated [85]. Moreover, comparable to T cells, NKT-like cells have shown higher sensitivity to stimulation with PMA and ionomycin [86]. It was also reported to increase IL-6 and TNF-α upon TCR stimulation [87]. The production of pro-inflammatory cytokines is also triggered by other immune activation signals, such as CD3 stimulation [88], the interaction of the cell adhesion molecule CD2 (LFA-1) [31], or the presence of infectious microorganisms, such as *Leishmania donovani* [89]. 

NKT-like cells are strong IFN-γ and TNF-α producers. Cytokine production is strongly stimulated by IL-15 and IL-23 and, to a lesser extent, by IL-2, IL-12, and IL-18. Additionally, anti-CD137, anti-CD3, and anti-CD2 also significantly increase the production of proinflammatory cytokines.

### 4.3. Homing and Chemokine Production

The chemotaxis of NKT-like cells is poorly understood. Virus-infected cord blood-derived mast cells (CBMC) produced CCL3, CCL4, and CCL5, among others. It was shown that supernatants from infected CBMCs selectively induced the migration of CD56+ T cells, almost two times higher than CD8+ T cells [90]. Additionally, CD56+ T cell migration was inhibited by a CCR1/CCR5 antagonist. CD56+ T cells expressed CCR5, but little CCR1, suggesting that mast cells may recruit CD56+ T cells via CCR5. Interestingly, NKT-like cells also produced higher levels of CCL4 [84], and it was demonstrated that CD56+ T cell supernatants promoted the production of CCR5 ligands against viral infection [91].

Several CC and CXC chemokines that are active toward neutrophils and monocytes were secreted by CD8+ NKT-like cells upon Fas engagement and upon TLR stimulation [87,92]. It was also shown that NKT-like cells might be recruited via the CX3CL1-CX3CR1 axis [93]. 

Aside from the limited studies, it was postulated that mast cells may considerably contribute to the attraction of NKT-like cells, possibly via CCR5, and that NKT-like cells contribute to the recruitment of other immune cells via Fas or TLR engagement. In addition to CCR5, CX3CR1 may also be crucial for the recruitment of NKT-like cells.

### 4.4. Effector Function

In addition to cytokine and chemokine production, NKT-like cells are also strong producers of perforin and granzyme B [80,94,95]. It was proposed that CD56 expression may possibly be an indication of effector T cells in light of the greater cytotoxicity reported for CD56+CD8+ T cells [6,46]. 

CD56+ T cells have potent cytotoxicity against Raji cells, a cell line sensitive to T cells, suggesting that NKT-like cells maintain the classical MHC-mediated cytotoxicity of CD8 T cells [84]. It was shown that the KIR+CD56+ T cell subset lysed cancer cells and CMVpp65-pulsed target cells in a dual KIR-dependent and TCR-dependent manner [7]. Since the K562 cell line does not express MHC molecules, it is vulnerable to NK cell activity. Similar to NK cells, the K562 cell line is also susceptible to CD56+ T cells, but to a lesser extent [96]. HLA class I-mediated recognition by inhibitory receptors may serve as a safeguard against imbalanced cytokine production and the killing by T cells that are activated independently of their TCR. The expression levels of HLA-A, -B, -C control the innate immunity through the engagement of inhibitory KIR on NK and presumably on NKT-like cells. MHC class Ib molecules HLA-E are orthologous to H-2-Qa1 as recognized by NK inhibitory receptors in mice [97]. HLA-E was demonstrated to be the ligand for members of the NKG2A/C-CD94 NK receptor family [98,99]. NKT-like cells also express the NKG2A/C-CD94 heterodimers that recognize the nonclassical MHC-Ib molecule, HLA-E in humans, and Qa1 in the mouse [100]. NKG2A has recently become known as a regulatory checkpoint for CD8 T cells [101]. Considering the receptor repertoire of NKT-like cells, antigens coupled to non-polymorphic MHC-E via NKG2A/C-CD94 receptors and classical polymorphic MHC-A, B, and C via inhibitory/activating KIR might control the NKT-like cells effector function.

MHC-unrestricted cytotoxicity by αβ TCR and γδ TCR T cells can be induced after being exposed to high quantities of IL-2. A potential molecule linked to or contributing to MHC-unrestricted cytotoxicity has been identified as NKR-P1 (CD161) [102]. In rats, αβ TCR T cells expressing low levels of NKR-P1 are incapable of MHC-unrestricted activity and reverse antibody-dependent cellular cytotoxicity (rADCC); although, both competencies can be developed when those cells are cultured with IL-2 in long-term cultures. 

NKG2D receptor activation enhances NK cell cytotoxicity while co-stimulating TCR signaling in T cells. It was proposed that NKG2D triggering, most likely through DAP10-mediated signaling, was responsible for the majority of the MHC-unrestricted cytotoxicity of activated and expanded CD8+ T cells [103], and it demonstrated a higher killing potential of CD3+CD56+ CIK cells against the A549 lung cancer cell line mediated by CD137 [79]. Curiously, NKG2D expression was elevated on CD3+CD56+ CIK cells derived from CD137-CIK cells. Newly obtained CD8+CD56+ T cells increased cytolytic activity toward both Raji (NK-resistant) and K562 (NK-sensitive) cells, which was successfully reduced by the perforin inhibitor (concanamycin A) [104]. Brefeldin A, an inhibitor of the Fas ligand, as well as partially an inhibitor of perforin, substantially reduced their increased cytolytic activity against HUVECs. 

Moreover, it was reported in mice that CD8+ NKT-like cells could kill DC-bearing antigens and inhibit the activation of T cells [105]. Similarly, it was shown that CD8+ NKT-like cells could kill antigen-dependent myeloid-derived suppressor cells (MDSC) in a granzyme B-dependent manner [106]. Additionally, it was demonstrated that CD56 cross-linking was sufficient to promote the production of several humoral factors [71]. An association between complement activation and the functional regulation of NKT-like cells has also been suggested since a variety of complement receptors and regulators, including CR3, C3aR, C5aR, C5L2, CD46, and CD55, are expressed by human NKT-like cells [107]. 

Although the activating and inhibitory mechanisms that control NKT-like cells are not well understood, recent research has shown that these cells are essential effectors that can function in both innate and adaptive ways. 

### 4.5. Resistance to Fas-Mediated Apoptosis

In the context of immune homeostasis, the process of program cell death or apoptosis is essential to control and suppress the activity of immune cells when it is no longer needed. Therefore, the deregulation of death receptor signaling, by either permitting too much or too little apoptosis, can result in autoimmune illnesses and have an impact on disease control. TNF family is the largest known family of “death receptors”, including TNF receptors (TNF-R), Fas (CD95), and dead receptors (DR4/5), and their ligands are all in the TNF-α family, including TNF-α, FasL, and TRAIL, respectively [42]. The presence of these dead receptors/ligands occurs not only in immune cells but also in other cells; indeed, tumor cells have mechanisms to counteract the immune response via the downregulation of the expression of dead receptors or by increasing the production of dead receptor ligands. 

The Fas/FasL system has at least two unique physiological roles in the peripheral immune compartment [108]. First, FasL is one of the primary effector pathways that activate T cells and natural killer (NK) cells that are used to attack target cells, along with granzyme/perforin secretion. Second, the expression of Fas and FasL is crucial for the control of immunological reactions, and it is crucial for the immune response’s dampening. Resting peripheral blood T lymphocytes are activated and upregulated for the Fas receptor and FasL upon the stimulation of the CD3/TCR complex. Nevertheless, during the initial phases of an immune response, they do not experience Fas-mediated apoptosis. T cells become vulnerable to Fas crosslinking and experience apoptosis after a period of either chronic stimulation or reactivation through the T cell receptor (TCR).

Remarkably, it was reported that CIK cells acquire resistance to Fas-mediated apoptosis [109]. In addition, CIK cells express increased levels of Fas, while the in-culture engagement of its ligand does not inhibit their cytotoxicity. Additionally, CIK cells also express FasL on their surface, and higher levels of its soluble form were found in the supernatants of cultures. Protein synthesis is necessary for CIK cells to withstand Fas ligation, and this resistance may develop as a result of the in vitro selection of Fas-resistant cells. Such resistance might be explained by the up-regulation of numerous anti-apoptotic genes, such as cFLIP, Bcl-2, Bcl-xL, DAD1, and survivin. Despite studies on NKT-like cells, which have shown a large amount of soluble FasL produced, their cytotoxic activity does not seem to be dependent on this pathway [82]. On the other hand, as stated above, NKT-like cells may take advantage of this pathway to recruit other immune cells. It was also demonstrated that CD56+ effector γδ T cells have an increased resistance to the Fas ligand and chemically induced apoptosis [110]. Moreover, Ugolini et al. demonstrated that a fraction of memory-phenotype CD8+ T cells that express IL-2Rβ is specifically driven to accumulate in vivo upon the recognition of MHC-I molecules by inhibitory NKR [30]. This points to an MHC-I-dependent mechanism that aids in the survival of memory CD8+ T cells.

**Figure 3:**
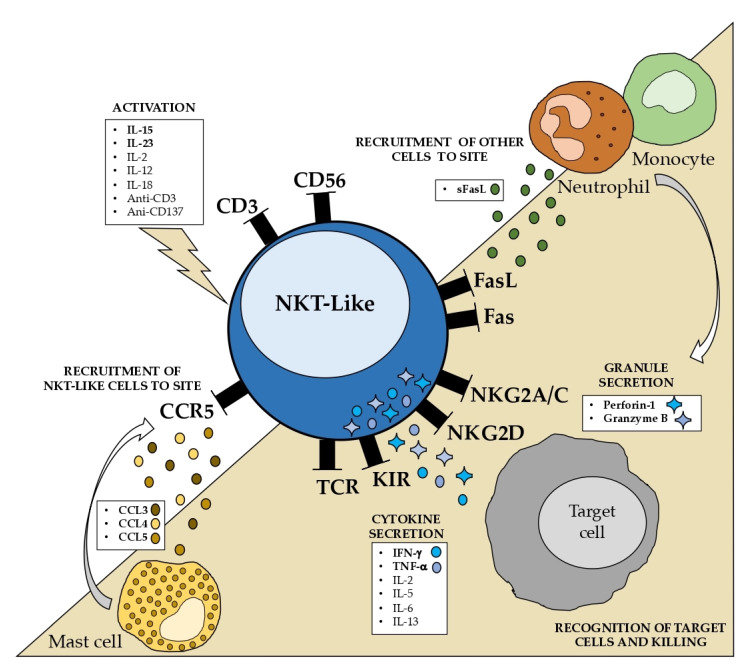
Illustration of the properties of NKT-like cells. NKT-like cells become activated when exposed to IL-15 and IL-23, but less so when exposed to IL-2, IL-12, IL-18, anti-CD3, and anti-CD137. IFN-γ and TNF-α are strongly produced in response to the majority of these molecules, and NKT-like cells also secrete IL-2, IL-5, IL-6, and IL-13. Mast cells secrete cytokines (CCL3, CCL4, and CCL5) attract NKT-like cells to the injured site. On the other hand, the production of soluble FasL may trigger the recruitment of additional immune cells such as neutrophils and monocytes. The effector function of NKT-like cells is regulated by the recognition of target cells through the TCR, KIR, NKG2A/C, and NKG2D receptors. When activated, NKT-like cells also release granules, such as perforin-1 and granzyme B, which are the path of choice to kill target cells. TCR—T cell receptor; KIR—killer-cell immunoglobulin-like receptor; sFasL—soluble FasL.

The state of our understanding of the properties and mechanisms underlying the NKT-like cell function is summarized in Figure 3. Some well-known characteristics, such as the increased cytotoxic capability without MHC restriction and the resistance to apoptosis, are particularly noteworthy. NKT-like cells should be considered separately from CD56- T cells and NK cells to completely comprehend the role of these cells in pathological circumstances.

## 5. NKT-like Cells: Role in Disease

The fact that the number of NKT-like cells rises with aging points to a critical function for these cells in the regulation of age-related disorders. Given their cytotoxic and cytokine production properties, it is feasible that NKT-like cells might play a significant role in the prevention or treatment of disease by removing infected or malignant cells. On the other hand, the accumulation of NKT-like cells may be harmful to the homeostasis of the immune system, resulting in chronic inflammation and autoimmune disorders.

### 5.1. Inflammation

NKT-like cells are strong producers of pro-inflammatory cytokines and, as such, may contribute to the support of inflammatory diseases. The frequency of NKT-like cells in the peripheral blood was found to increase in sarcoidosis patients, where there is a formation of granulomas that emerge preferentially in the lung or lymph nodes. However, no differences were observed in bronchoalveolar lavage fluid (BALF) [111]. The frequency of CD8+ NKT-like cells has been found to increase in the BALF of hypersensitive pneumonitis and chronic obstructive pulmonary disease (COPD) patients [112,113,114,115]. In COPD patients, NKT-like cells were found to increase in saliva samples and decrease in the peripheral blood, compared with healthy donors, indicating that circulating NKT-like cells might contribute to the in-situ inflammation of the airways. In Behçet’s uveitis patients, it was suggested that the infiltration of cytotoxic NKT-like cells in the intraocular area may participate in chronic recurrent uveitis and obliterate retinal vasculitis [104,116]. NKT-like cells are predicted to contribute to the increased cytokine milieu that is typical of inflammation, namely when their effector activity is dysregulated. Thus, targeting NKT-like cells, in addition to T and NK cells, could be considered a way to lessen the inflammatory milieu in these inflammatory-related disorders. 

### 5.2. Infection

Since it is known that NKT-like cells can kill cells that are infected with pathogens, it is important to determine whether they play a significant role in infectious diseases. According to studies on liver infections, NKT-like cells may contribute to the control of several strains of the hepatitis virus. Patients with the hepatitis B virus (HBV) showed elevated levels of circulating NKT-like cells, and IL-17 associated with the HBV mRNA level, which decreases along with recovery [117]. A reduced response to the HBV vaccine in individuals with a poor activation of circulating NKT-like cells was observed [118]. Likewise, CD16+ NKT-like cells were found to be significantly increased in the peripheral blood of hepatitis E virus (HEV) patients, and recovered patients had a higher expression of the cytotoxic receptors NKp44 and NKp46, and the activating receptor NKG2D [119,120]. In Hepatitis C Virus (HCV) infection, the increased activation of NKT-like cells at the baseline is associated with spontaneous recovery, whereas the inhibited activity culminates with persistent infection, possibly due to the increased expression of the inhibitory receptor NKG2A [121]. The expression of the NKG2D receptor was implicated in the response to treatment [122]. In HBV and HEV infection, there are no data on liver NKT-like cells in HCV, the CD56+ αβ T cells and iNKT cells which were found to be depleted from the livers of patients with mild chronic HCV infection [123]. Circulating NKT-like cells seem to be critical players in controlling systemic infection, which may not be applied to liver NKT-like cells. Also, in human immunodeficiency virus (HIV) infection, the percentage of activated IFN-γ+CD107a+ NKT-like cells is correlated with the viral load [124]. Moreover, the long-term non-progressor patients exhibited a higher frequency of activated NKT-like cells compared to primary and chronic HIV-infected patients. In vitro studies demonstrated that supernatants collected from CD56+ T cell cultures inhibited HIV-1 infection and replication [91]. A possible role for NKT-like cells in controlling the Chikungunya virus in the convalescent stage after the infection has been postulated [125]. 

Additionally, NKT-like cells have been implicated in bacterial and parasitic infections. In pulmonary tuberculosis, it was possible to discriminate between patients with active disease and those who were latently infected by analyzing the IFN-γ produced by circulating NKT-like cells [126]. In recurrent furunculosis, increased levels of these cells in the peripheral blood of patients were also observed [127]. There are also reports suggesting a role for NKT-like cells in controlling *Leishmania donovani* parasitic infection. In visceral leishmaniasis, it was shown that CD4+ NKT-like cells, not CD8+, migrated to the site of the infection [89]. Those cells are similar to regulatory T cells (CD25+Foxp3+) and produce IL-10, meaning that a suppressive microenvironment that may support the infection could be created. On the other hand, CD8+ NKT cells are more prone to being protective. In cutaneous leishmaniasis, caused by *Leishmania (Viannia) braziliensis* (Lb), a higher frequency of CD8 T, NK, and NKT-like cells, contrary to CD4 T cells, in cultures of PBMC collected from patients before treatment and exposed to Lb antigens was observed [128]. CD8+ NKT-like cells appear to be protective in both cases of visceral or cutaneous leishmaniasis, in contrast to CD4+ NKT-like cells. 

In general, individuals with infectious diseases have higher levels of circulating NKT-like cells, and their activation is somehow related to an improved capacity to manage the infection.

### 5.3. Pregnancy

A dominant role for innate immunity rather than adaptive immunity has been suggested in the immunoregulation of pregnancy [129]. Menstrual blood and peripheral blood appear to have the same histological characteristics, but menstrual blood contains a different set of NKT-like subsets with different cytokine profiles. Women with unexplained infertility had a lower frequency of menstrual blood NKT-like cells than the fertile and unexplained recurrent spontaneous abortion (URSA) women [130]. It was also found that higher levels of circulating CD16+ NKT-like cells are linked to higher rates of successful pregnancy and live births [131]. Interestingly, in cases of recurrent pregnancy loss or implantation failure, the usage of intravenous immunoglobulin (IVIG) therapy is associated with an increased number of blood NKT-like cells, improving the likelihood of successful pregnancy [132]. In contrast, when compared to women who had succeeded in vitro fertilization (IVF), the NKT-like cell population in the follicular fluid of unsuccessful gestation was noticeably higher [133]. It was also proposed that CD16+ NKT-like cells, IFN-**γ**, and IL-2 could be utilized as indicators to estimate the likelihood of miscarriage in underexplained recurrent miscarriage patients [35]. Recently, it was also suggested that highly cytotoxic CD56+ γδ T cells are inhibited by the expression of the PD-1 receptor, which prevents overreaction at the feto–maternal interface [134]. The rate of PD-1 expression by CD56+ γδ T cells increased throughout the first trimester and then returned to normal levels afterward. Deficient inhibition of these potentially cytotoxic T cells may lead to pregnancy complications. Women with thyroid autoimmunity (TAI) have an increased risk of infertility, recurrent miscarriage, and IVF failure [135]. The analysis of PBMC from TAI women revealed an increase in the Th1-like response of NKT-like cells with increased cytotoxicity, and this may also contribute to complications during gestation. 

These findings suggest that NKT-like cells play a crucial role in a healthy pregnancy. As a result, the monitoring of pregnant women must include the analysis of circulating NKT-like cells.

### 5.4. Transplantation

Similar to a successful pregnancy, successful transplantation also depends on tolerance. Similar to T and NK cells, NKT-like cells may kill non-self-targets, contributing to organ rejection. In the context of liver transplantation, intrahepatic NKT-like cells, which represent about half of the T cells in the liver, were found to be drastically reduced during severe graft rejection, in contrast with T cells, suggesting that the tolerogenic milieu of the liver is significantly influenced by NKT-like cells [136].

In-depth research on lung transplant rejection and bronchiolitis obliterans syndrome (BOS) has been conducted by Hodge and collaborators. Unsuccessful transplant patients had a significantly higher frequency of NKT-like cells that produced IFN-γ, TNF-α, IL-2, IL-17, granzyme B, and perforin [137]. Therefore, it was claimed that current immunosuppressive protocols are insufficient for reducing the number of NKT-like cells and the release of pro-inflammatory mediators that are known to be linked with graft rejection. BOS is associated with increased cytotoxic/pro-inflammatory CD8+ T, NKT-like, and NK cells in the small airways [138]. After lung transplantation, an immunosuppressive medication frequently fails to stop BOS, a condition mostly affecting the small airways. Pro-inflammatory gene expression is increased and decreased by histone acetyltransferases (HATs) and histone deacetylases (HDACs), respectively. Following lung transplant, HDAC2 is reduced in CD8+ T and NKT-like pro-inflammatory cells [139]. Options for therapy that increase HDAC2 levels may enhance graft survival. Additionally, after lung transplantation, BOS is associated with a reduction in SIRT1 in proinflammatory T, NK, and NKT-like cells in the peripheral circulation [140]. Thus, contrary to liver transplantation, increased NKT-like cells were associated with graft rejection in lung transplantation. 

### 5.5. Autoimmunity

The dysregulation of the immune system can cause serious damage to the host, leading to autoimmune disorders. The lack of control of cytokine production, and the cytotoxic activity of immune cells leads to chronic inflammation which is a hallmark of these pathological conditions. Even though NKT-like cells remain largely underexplored in this context, some investigations suggest a possible role for these cells in systemic autoimmunity. 

In rheumatoid arthritis (RA) and Th1-type autoimmune disease, studies are controversial. A decrease in circulating NKT-like cells was shown in RA patients [141]. However, other studies suggested that the accumulation of CD56+ T cells in the peripheral blood of RA patients may be prejudicial [10]. They claim, given the loss in the expression of CD28 and correlation with the acquired expression of CD56, that CD56+ T cells are the result of processes that can contribute to triggering RA. In juvenile idiopathic arthritis (JIA), low levels of peripheral NKT-like cells were observed in patients with the systemic form when compared to the controls [142]. In comparison with peripheral blood, the synovial fluid contained more NKT-like cells and higher concentrations of perforin, granzyme B, and TNF-α. In JIA polyarticular patients, the frequency of NKT cells was implicated in the response to etanercept since patients who responded exhibited a lower frequency of NKT-like cells after treatment. 

Systemic sclerosis (SSc) involves connective tissue and microvasculature. Patients with advanced disease exhibited higher levels of circulating CD8+ T cells, NK cells, and NKT-like cells [143]. The maintenance of inflammation in Systemic Lupus Erythematosus (SLE) patients may be aided by increased granzyme B secretion from NK and NKT-like cells in active SLE patients, which is further exacerbated by circulating IL-15 [144]. In primary Sjögren’s syndrome (pSS), the patients’ peripheral NKT-like cells were also found to be significantly decreased compared with the healthy controls [93]. Contrarily, in the labial salivary gland, these cells were found at a higher frequency. Another study supports the reduced frequency of NKT-like cells in pSS, suggesting that they may play a role in the pathophysiology of the disease [145]. Furthermore, lower NKR+ T cell counts in inflamed mucosa were seen in ulcerative colitis (UC), and they were found to be inversely correlated with the degree of inflammation, indicating the protective role of these cells in UC [54,146]. 

Even though NKT-like cells may play a protective role occasionally, these findings imply that, in general, NKT-like cells are involved in the persistence of autoimmune diseases.

### 5.6. Neurological Disorders

Neurodegenerative disorders and inflammation usually coexist [147]. It is, therefore, possible that NKT-like cells are involved in these pathologies. When compared to healthy individuals, Parkinson’s disease (PD) patients tend to have higher peripheral levels of NKT-like cells, which significantly decrease after one and two years of treatment [147,148]. Furthermore, it was demonstrated that elevated levels of the soluble VLA4 ligand VCAM1 in the plasma of Parkinson’s patients are related to the decreased expression of VLA4 in peripheral NKT-like cells [148]. Moreover, although the frequency of peripheral blood NKT-like cells was found to be unaltered in Alzheimer’s disease (AD), vascular dementia (VD), and frontotemporal dementia (FTD) patients, NKT-like cells present in cerebrospinal fluid (CSF) were shown to be more prevalent in all stages of AD, VD, and FTD [149,150]. In fact, a direct link was shown between pro-inflammatory NKT-like cells and Alzheimer’s disease (AD) [151]. A function for PD-L1 expression in these cells was also reported [152]. In AD, as suggested by Solana et al., microglia phagocytes amyloid-β attract NK cells into the brain, generating proinflammatory cytokines that cause neurodegeneration [153]. We could speculate that NKT-like cells are impacted by the same process, thus contributing to neuroinflammation.

Currently, NKT-like cells are a negligible research topic in autoimmune neurological disorders. There is no evidence to suggest that these cells have a significant role in Myasthenia Gravis or Guillain–Barré syndrome [154]. In MS patients, as in AD, the expansion of NKT-like cells was observed in the CSF and associated with disease activity [155]. In addition, IFN-β treatment reduced the proportion of NKT-like cells in CSF, while untreated RRMS and neuromyelitis optica syndrome disease (NMOSD) had higher levels when compared to healthy controls [156]. Furthermore, NKT-like cells were found to be elevated in the CSF of patients with chronic inflammatory demyelinating polyneuropathy (CIDP), together with CD8 T cells, indicating that these cells may promote inflammatory neuropathy [157]. Due to the potential of NKT-like cells to produce high amounts of pro-inflammatory cytokines and their cytotoxic potential, research into the role of these cells in neuroinflammation-related diseases may contribute to clarifying the inflammatory state that is found in the peripheral and central nervous systems.

Growing evidence that psychosis is a type of autoimmune disease strongly links immunological dysfunction to the pathogenesis of neuropsychiatric disorders. Significant NK cell impairment in first-episode psychosis, schizophrenia, and bipolar disease supports the idea that innate immunity plays a key role in these pathological situations [158,159]. It has been suggested, for instance, that circulating IFN-**γ**-producing NKT-like cells may be involved in controlling the pathogenesis of drug-resistant epilepsy [160]. Given their intrinsic innate-like function, we postulate that NKT-like cells may be involved in psychosis, despite the lack of knowledge on their involvement in these disorders.

### 5.7. Cancer

Knowing that cytotoxic lymphocytes have potent anti-tumor activity, the current research attempts to determine whether these cells can be assessed and tracked to predict disease outcomes and to use these cells for novel immunotherapy strategies. Several authors have discussed the potential contribution of NKT-like cells to hematologic and solid tumors. For instance, progressive chronic lymphocytic leukemia (CLL) patients had lower levels of peripheral CD16+ NKT-like cells, which were inversely correlated with suppressor Treg cells [161,162]. We reported a lower frequency of NKT-like cells and a rearranged receptor repertoire in chronic myeloid leukemia patients [163]. Additionally, others suggested that NKT-like cells may offer protection against B cell-CLL and acute myeloid leukemia [164,165].

NKT-like cells may in some way contribute to an effective anti-tumoral immune response. The significance of NKT-like cells in hepatitis prevention and treatment was previously highlighted [121]. Liver inflammation and infection may play a role in the emergence of hepatocellular carcinoma (HCC), and NKT-like cells may also be crucial in this scenario [166]. In fact, it is currently known that CD56+ T and NK cells can destroy HCC cells, although it is likely that they are unable to exert their anti-tumor activity in vivo because they are reduced in the patient’s livers and are scarce in those with metastatic disease [167,168]. Next, it was also reported that circulating NKT-like cells are dysfunctional in HCC patients due to the impaired production of TNF-α and IFN-γ [169]. In melanoma patients, the decreased frequency of circulating PD-1+CD56+ T cells was associated with improved survival [170]. Low levels of peripheral NKG2D+ and NCR+ NKT-like cells were found in colorectal cancer (CRC), while high levels of CD16+ NKT-like cells were independently linked to a shorter disease-free survival time [171,172]. In gastric cancer (GC), it was shown that a reduced frequency of NKT-like cells expressed activatory receptors and impaired anti-tumoral activity in malignant tissue, which was correlated with shorter survival rates [173]. Moreover, a higher frequency of infiltrating NKT-like cells was present in intestinal cancers when compared to pancreato-biliary tumors, which was also associated with a better clinicopathological outcome [174]. Also, in the early stages of lung cancer (LC), patients had considerably higher numbers of circulating NKT-like cells [175]. Overall, there is evidence to suggest that NKT-like cells may be important for cancer prevention and management; however, because NKT-like cells are so highly variable and tissue-dependent, it is important to keep in mind that some subpopulations may have pro-tumor effects. Therefore, to distinguish between favorable and negative prognostic markers, a more comprehensive investigation must be performed when evaluating the predictive value of NKT-like cells.

It was demonstrated that previously cryopreserved cord-blood mononuclear cells could be expanded ex vivo to obtain efficient activated NKT-like subsets, suggesting a possible use of these cells in adoptive cellular immunotherapy [176]. The assumption that NKT-like cells must be investigated in that context is mostly supported by the various clinical trials that have been undertaken on cancer patients who received CIK cells. Since the terminally developed CD3+CD56+ CIK subpopulation resembles NKT-like cells in vivo [177]. A viable strategy for cancer immunotherapy using CIK cells was demonstrated, with little to no graft versus host disease (GvHD) damage [178]. More work has recently been conducted toward enhancing the antitumoral efficacy of CIK cells, including the use of immune checkpoint inhibitors (ICI) and chimeric antigen receptors [179,180,181]. For example, the combination with immune checkpoint inhibition improved CIK cytotoxicity against kidney cancer cells [182]. It was also demonstrated that the anti-PD-1 blockade rescued dysfunctional NKT-like cells in HCC [169]. In addition, in melanoma patients, it was suggested that the evaluation and monitoring of PD1+ NKT-like cells might help to predict the response to anti-PD-1 therapy [170]. Considering the similarities between NKT-like and CIK cells, we might speculate that if NKT-like cells are activated and expanded ex vivo, they might exhibit the same qualities as CIK cells, which could eventually be improved when combined with ICI.

### 5.8. Other Disorders

Heart diseases are another area of study for NKT-like cells. In contrast to controls, the blood accumulation of CD8+ NKT-like cells was seen in patients with acute coronary syndrome and stable angina [183]. These cells produce higher levels of IFN-γ than their CD8+CD56− counterparts. Additionally, CD8+ NKT-like cells exhibit decreased CD28 expression and apoptotic resistance. According to Bergström et al., the atherosclerotic process is aided by these cells’ production of IFN-γ [183].

When comparing patients with diabetes to the control group, the frequency of circulating NKT-like cells was also considerably higher in pre-diabetes patients with atherosclerosis-inducing dysglycaemic illness (diabetes type 2) [184]. Pre-diabetic individuals showed high levels of NKT-like cells expressing granzyme B and perforin in comparison with the diabetic and control groups. It was proposed that diabetes type 2 etiology and severity may be influenced by CD56+ T cells expressing NKG2D, particularly by the production of IL-17 [185]. Interestingly, IFN-γ-expressing CD3+CD4+CD56+ NKT-like cells were associated with an increased incidence of coronary diseases [25].

NKT-like cells shouldn’t be disregarded in light of the information presented above since, as demonstrated in Table 1, they may be implicated in the control of a number of clinical diseases. NKT-like cells generally appear to add to the severity of inflammatory and autoimmune illnesses; however, in the situation of infection or cancer, they seem to be crucial in the removal of infected or transformed cells.

## 6. Future Perspectives

Even though NKT-like cells are potent immune effectors with a few unique characteristics, including high cytotoxic activity and resistance to apoptosis, their significance in health and disease is still poorly understood and requires more research. It is critical to comprehend if these cells serve as favorable or unfavorable prognostic indicators in aging, particularly in age-related disorders. The quantification and characterization of these cells might serve as a cutting-edge indicator of individual immune health. Additionally, exploring the mechanisms that can control their killing activity in different contexts may, therefore, result in innovative therapeutic alternatives in a wide range of diseased settings.

## 7. Conclusions

The definition of NKT-like cells (CD3+CD56+) was raised to clearly distinguish those cells from the classical NKT family. NKT-like cells represent highly differentiated, conventional, or unconventional T cells that co-express the CD56 molecule and other NKR and recognize antigens in a “CD1d-independent” manner. Even though NKT-like cells are a very heterogeneous population, most of them co-express both the αβ TCR and CD8 molecules, and they all have at least three things in common: the mature phenotype, NKR acquisition, and granular morphology. 

The origin of NKT-like cells is still unclear. The most plausible explanation is that these cells differentiate outside of the thymus in response to ongoing antigen exposure, which is supported by the higher frequency of these cells in organs such as the liver, gut, and bloodstream. The fact that the frequency of peripheral NKT-like cells rises with aging also supports the fact that ongoing antigen exposure may prompt NKT-like cell differentiation. Moreover, according to certain proposals, the expression of CD56 and other NKR may indicate a remodeling mechanism that promotes successful aging rather than a useless byproduct of aging.

It has been shown that IL-15, IFN-γ, anti-CD3, and anti-CD137 stimulate the growth and proliferation of NKT-like cells. An increase in the acquisition of CD56 expression by T cells in PBMC in vitro co-cultures suggests that NKT-like cells may proliferate in vivo through exposure to cytokines or via cell-cell contact. It was proposed that NKT-like cells have a substantial role in the production of IFN-γ and TNF-α, which is strongly increased by IL-15 and IL-23, and to a lesser extent by IL-2, IL-12, and IL-18, as well as anti-CD3 and anti-CD137. In addition to the few examples of research on the chemotaxis of NKT-like cells, it is hypothesized that these cells play a significant role in attracting neutrophils and other immune cells. It is also likely that mast cells play a role in attracting NKT-like cells via CCR5. 

Moreover, NKT-like cells are not restricted to an MHC-mediated effector function. The acquisition of NKR by T cells, including CD56, confer additional mechanisms to these cells as the non-MHC mediated cytotoxicity. The expression of MHC-Ia and Ib molecules on target cells can inhibit or activate the function of NKT-like cells, given the expression of inhibitory/activating KIR and NKG2A/C, respectively. NKT-like cells retain some characteristics of classical T cells, mainly the expression of the CD8 molecule, and they can kill targets through classical MHC recognition. The resistance to Fas-mediated apoptosis is another intriguing feature of NKT-like cells. On the other hand, whereas FasL is expressed by NKT-like cells, there is no correlation between its activity and cytotoxicity. 

The fact that NKT-like cell counts increase with age suggests that these cells play a crucial role in the control of disorders associated with aging. It is conceivable that NKT-like cells could be crucial in the prevention or treatment of disease by eliminating infected or tumor cells due to their cytotoxic and cytokine-producing abilities. Additionally, NKT-like cells seem to be an important player in successful pregnancy and transplantation. The accumulation of NKT-like cells, on the other hand, may be detrimental to the immune system’s equilibrium and result in autoimmune diseases and chronic inflammation.

## Figures and Tables

**Table 1:** Summary of previous studies evaluating the role of NKT-like cells in disease.

Disease Type	Disease	NKT-like Cells Findings	Beneficial	Reference
Inflammation	Hypersensitive Pneumonitis	•Increased in BALF	No	[112]
	Sarcoidosis	•Equal in BALF	---	[111]
		•Increased in PB	No	[111]
	COPD	•Increased in BALF	No	[113]
		•Increased in saliva	No	[114]
		•Decreased in PB	No	[114]
	Behçet’s uveitis	•Increased in the intraocular area	No	[116]
Infection	Hepatitis C virus	•Decreased in Liver	---	[123]
		•Increased in PB	Yes	[121]
	Hepatitis B virus	•Increased in PB	Yes	[117]
	Hepatitis E virus	•Increased in PB	Yes	[119]
	Human Immunodeficiency virus	•Increased IFN-γ production in PB	Yes	[124]
	Chikungunya virus	•Increased in PB	Yes	[125]
	Recurrent furunculosis	•Increased in PB	Yes	[127]
	Pulmonary tuberculosis	•Increased IFN-γ production in PB	Yes	[126]
	Leishmaniasis	•Increased CD8+ at the site of infection	Yes	[128]
Pregnancy	Unsuccessful pregnancy	•Increased in follicular fluid	No	[133]
		•Increased CD16+ in PB	Yes	[131]
	Unexplained recurrent miscarriage	•Increased activation in PB	No	[35]
	Unexplained infertility	•Decreased in MB	No	[130]
	IVIG therapy	•Increased in PB	Yes	[132]
	Thyroid Autoimmunity	•Increased Th1-like in PB	No	[135]
Transplantation	GvH disease lung	•Increased in PB	No	[140]
	BOS after transplantation	•Increased in small airways	No	[138]
	GvH disease liver	•Decreased in Liver	Yes	[136]
Autoimmunity	Rheumatoid Arthritis	•Increased in PB	No	[10]
	Juvenile idiopathic arthritis	•Decreased in PB	No	[142]
		•Increased in synovial fluid	No	[142]
	Systemic sclerosis	•Increased in PB	No	[143]
	Systemic Lupus Erythematosus	•Increased in PB	No	[144]
	Sjögren’s syndrome	•Decreased in PB	No	[145]
		•Increased in labial salivary gland	No	[93]
	Ulcerative Collitis	•Decreased NKR+ in inflamed mucosa	No	[54]
Neurological disorders	Parkinson’s disease	•Increased in PB	No	[148]
	Alzheimer’s disease	•Increased in CSF	No	[150]
	Vascular dementia	•Increased in CSF	No	[150]
	Frontotemporal dementia	•Increased in CSF	No	[150]
	Multiple Sclerosis	•Increased in CSF	No	[155]
	Chronic inflammatory demyelinating polyneuropathy	•Increased in CSF	No	[157]
	Epilepsy	•Increased IFN-γ production in PB	Yes	[160]
Cancer	Colorectal cancer	•Decreased NKG2D+ in PB	No	[171]
		•Increased CD16+ in PB	No	[172]
	Chronic lymphocytic leukemia	•Decreased in PB	No	[161]
	Chronic myeloid leukemia	•Decreased in PB	No	[163]
	Hepatocellular carcinoma	•Decreased in liver	No	[167]
	Lung cancer	•Decreased in PB	No	[175]
	Melanoma	•Increased PD-1+ PB	No	[170]
	Gastric cancer	•Decreased in malignant tissue	No	[173]
	Periampullary adenocarcinoma	•Decreased in malignant tissue	No	[174]
Others	Acute coronary syndrome	•Increased IFN-γ production in PB	No	[183]
	Pre-diabetes	•Increased in PB	No	[184]

Legend: PB—Peripheral blood; MB—Menstrual blood; NKR—Natural killer receptors; CSF—Cerebrospinal fluid.

## Data Availability

Not applicable.
